# Epibiont communities on mussels in relation to parasitism and location in the rocky intertidal zone

**DOI:** 10.1093/femsec/fiae101

**Published:** 2024-08-13

**Authors:** Katherine M Davis, Laura Wegener Parfrey, Christopher D G Harley, Keith Holmes, Olivia Schaefer, Alyssa-Lois Gehman

**Affiliations:** Biodiversity Research Centre, University of British Columbia, Vancouver, BC V6T 1Z4, Canada; Department of Botany, University of British Columbia, Vancouver, BC V6T 1Z4, Canada; Biodiversity Research Centre, University of British Columbia, Vancouver, BC V6T 1Z4, Canada; Department of Botany, University of British Columbia, Vancouver, BC V6T 1Z4, Canada; Department of Zoology, University of British Columbia, Vancouver, BC V6T 1Z4, Canada; Biodiversity Research Centre, University of British Columbia, Vancouver, BC V6T 1Z4, Canada; Department of Zoology, University of British Columbia, Vancouver, BC V6T 1Z4, Canada; Hakai Institute, PO Box 25039 Campbell River, BC V9W 0B7, Canada; Department of Zoology, University of British Columbia, Vancouver, BC V6T 1Z4, Canada; Biodiversity Research Centre, University of British Columbia, Vancouver, BC V6T 1Z4, Canada; Department of Zoology, University of British Columbia, Vancouver, BC V6T 1Z4, Canada; Hakai Institute, PO Box 25039 Campbell River, BC V9W 0B7, Canada

**Keywords:** cyanobacteria, host–bacteria interactions, intertidal

## Abstract

The factors shaping host–parasite interactions and epibiont communities in the variable rocky intertidal zone are poorly understood. California mussels, *Mytilus californianus*, are colonized by endolithic cyanobacterial parasites that erode the host shell. These cyanobacteria become mutualistic under certain abiotic conditions because shell erosion can protect mussels from thermal stress. How parasitic shell erosion affects or is affected by epibiotic microbial communities on mussel shells and the context dependency of these interactions is unknown. We used transplant experiments to characterize assemblages of epibiotic bacteria and endolithic parasites on mussel shells across intertidal elevation gradients. We hypothesized that living mussels, and associated epibacterial communities, could limit colonization and erosion by endolithic cyanobacteria compared with empty mussel shells. We hypothesized that shell erosion would be associated with compositional shifts in the epibacterial community and tidal elevation. We found that living mussels experienced less shell erosion than empty shells, demonstrating potential biotic regulation of endolithic parasites. Increased shell erosion was not associated with a distinct epibacterial community and was decoupled from the relative abundance of putatively endolithic taxa. Our findings suggest that epibacterial community structure is not directly impacted by the dynamic symbiosis between endolithic cyanobacteria and mussels throughout the rocky intertidal zone.

## Teaser

This study uses transplant experiments on rocky intertidal shores to understand the association between bacterial communities on mussel shells and shell erosion caused by cyanobacterial parasites.

## Introduction

Mussels are important intertidal foundation species. The microbial communities living on or in mussels play a central role in host health as well as nitrogen and carbon cycling (Pfister 2007, Heisterkamp et al. 2013, Pfister and Altabet 2019) in coastal ecosystems. The intertidal zone where mussels live is a heterogeneous habitat with abiotic gradients of temperature, salinity, nutrients, ultraviolet light, wave action and rainfall (Connell 1972, Helmuth and Hofmann 2001, Harley and Helmuth 2003). The position of a mussel within the intertidal zone can affect host physiology (Place et al. 2012) and biotic interactions (Paine 1974, Lubchenco 1980), all of which can influence the epibiotic microbial community. Characterizing the abiotic and biotic drivers of host–microbial associations in foundation species, like mussels, is critical for understanding the contribution of microbes to diverse physiological and ecological functions. It can also aid in predicting the potential outcomes of environmental change on host–microbial associations (Wilkins et al. 2019).

Mussels have long been a model of ecological zonation in the intertidal (Paine 1966, 1974) and are increasingly susceptible to mass mortality events (Harley 2008, Seuront et al. 2019) and other climate change-related stressors (Frölicher et al. 2018). Thus, natural populations of intertidal mussels represent a valuable system for studying host–microbial associations across heterogenous environmental gradients that are under heightened pressures from climate change. Previous studies have investigated the bacterial diversity in the internal tissues and fluids of mussels (Li et al. 2018, Vezzulli et al. 2018), especially in relationship to disease-causing agents (Li et al. 2019), because of their economic value and impacts on human health (Rubiolo et al. 2019). Less is known about the factors shaping the epibiotic shell bacterial community, which serves as an interface or protective cover between mussels and the abiotic environment.

Some constituents of the mussel shell microbial community, endolithic cyanobacteria, have attracted the attention of researchers because of the role these organisms play in host survivability. Endolithic cyanobacteria are parasitic microbes that bore into mussel shells causing decreased shell thickness and strength. The energetic costs to repair shell damage can compromise mussel growth, byssal attachment strength and increase mortality (Kaehler 1999, Zardi et al. 2009). The boring activity by endolithic cyanobacteria removes the dark, outer periostracum of mussel shells exposing the light gray prismatic layer. The lighter-colored, eroded shells reflect solar radiation, and the porosity of eroded shells also helps mussels retain more water compared with mussel shells with little erosion (Gehman and Harley 2019). Consequently, high levels of endolithic cyanobacteria infestation and resultant erosion reduce stressful heat gain and mussel mortality during high temperature events at low tide (Zardi et al. 2016, Gehman and Harley 2019). Thus, the parasitic shell-boring cyanobacteria become mutualistic in a context-dependent manner during intense thermal stress (Gehman and Harley 2019). There is some evidence that infestation by endolithic cyanobacteria increases with elevation in the intertidal zone because photosynthesis is enhanced by the prolonged exposure to light (Marquet et al. 2013). The relationship between endolithic cyanobacteria abundance, shell erosion and intertidal elevation in the northeastern Pacific is not well understood and the composition of endolithic cyanobacteria assemblages on mussel shells in this region have not yet been examined using molecular tools (Bower et al. 2002). This study characterized the cyanobacterial assemblages on shells of the California mussel, *Mytilus californianus*, using 16S rRNA amplicon sequencing. It further examined the relationship between live hosts, endolithic parasite identity, shell erosion and the overall epibiotic bacterial community across elevation gradients in the rocky intertidal zone at four sites in British Columbia, Canada.

Microbial colonization of mussel shells may be constrained by the selective filter of host biology. Host biology has been demonstrated to influence the microbial communities occupying internal compartments of diverse host species (Woodhams et al. 2020), as well as the protective outer layers of marine hosts like corals (Glasl et al. 2016). For mussels, the biochemical composition (Bers et al. 2006) and microtopographies (Bers et al. 2005) of an intact periostracum, or waste products excreted by the host (Pfister et al. 2014), can influence the microbial taxa that colonize the outer shell. For macroscopic epibionts such as barnacles, growth is significantly faster for individuals that settle on live mussels compared with empty shells, likely because of the increased nutrition derived from association with a living host (Laihonen and Furman 1986). Alternatively, mussel shells may be surfaces with limited microbial selectivity, colonized by communities whose taxonomic composition, growth and productivity are strictly constrained by abiotic factors (Palinska et al. 2017). Experimental manipulation is a necessary tool for determining the role of host biology and abiotic variation in shaping the epibiotic microbial composition on mussel shells.

In this study, we experimentally tested the hypothesis that live mussels influence the composition of epibiotic bacterial assemblages and modulate shell erosion caused by endolithic cyanobacteria. We transplanted minimally eroded pairs of live mussels and shucked mussel shells across an intertidal elevation gradient at four sites and characterized the epibiotic community 3 to 5 months later using 16S rRNA gene amplicon sequencing. We used killed, shucked mussels to represent shell substrate in the absence of host biological filtering. We predicted (i) that the shells of transplanted live mussels would be less susceptible to erosion by endolithic cyanobacteria; (ii) that high shell erosion, which can modify host physiology, shell microtopography (Zardi et al. 2009) and abiotic conditions experienced by the host (Zardi et al. 2016) and epibiont communities, would influence which bacteria colonize a mussel shell, and therefore be associated with unique taxa compared with mussel shells with less erosion; and (iii) that the bacterial community composition on live mussels would be less variable than on empty shells when exposed to different abiotic conditions because of the potential buffering capacity provided by a live biogenic habitat.

## Materials and methods

### Experimental conditions

**Figure 1.**
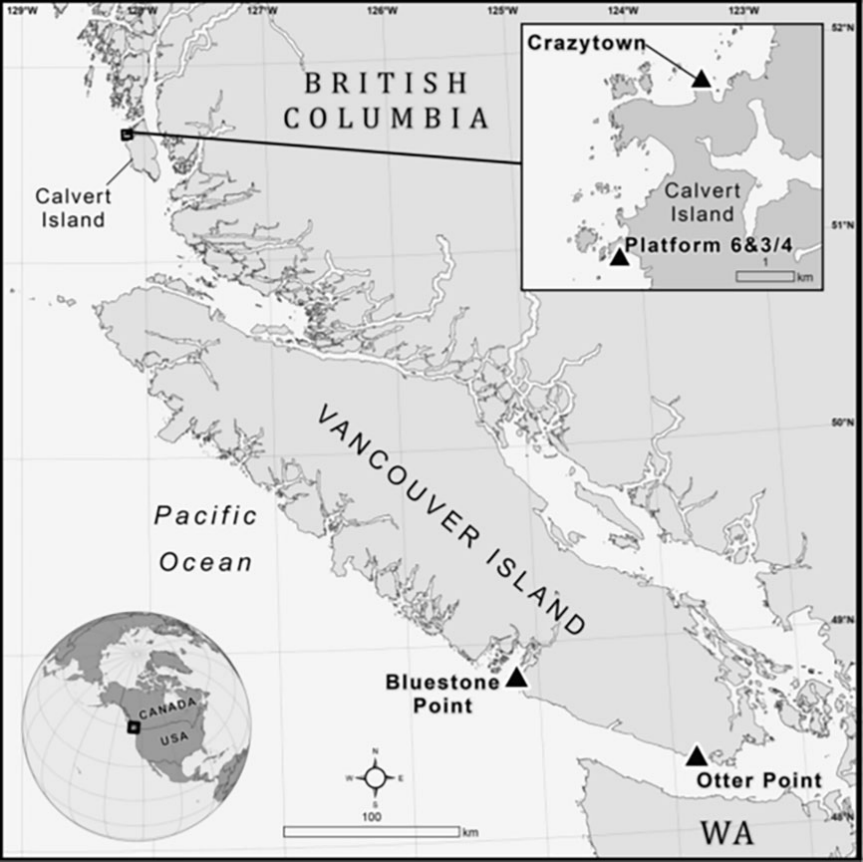
Map of experimental sites.

Mussel transplant experiments were conducted in 2017, at four sites around coastal British Columbia, Canada, which are dominated by the California mussel, *Mytilus californianus* (Fig. 1). Two sites were on Calvert Island near the Hakai Institute Ecological Observatory: one on the north side of the island (Crazytown, Table 1) and one on the west side of the island (Platform 6 ¾, Table 1). The other two sites were located on Vancouver Island, Otter Point in Sooke and Bluestone Point near the Bamfield Marine Sciences Centre (Table 1).

**Table 1.** Study locations, number of experimental transplants, key dates, and sample numbers.

						Transplant date	Microbiome sampling date	Microbiome samples analyzed		
Site	Region	Latitude	Longitude	Elevation range (m)	Initial experimental pairs (n)	2017	2017	Live (n)	Killed (n)	Unmanipulated controls (n)
Bluestone Point	Vancouver Island	48.82304	−125.1617	2.86–3.18	11, 10	28 April, 27–28 June	18 Sept	19	24	10
Otter Point	Vancouver Island	48.35714	−123.8231	1.44–3.13	11, 12	26–27 April, 21, 23–24 June	18 Sept	19	21	11
Crazytown	Calvert Island	51.66751	−128.1333	3.07–4.10	19	26–28 May	21 Sept	14	13	14
Platform 6 and 3/4	Calvert Island	51.63939	−128.1525	1.66–2.75	23	26–30 April	20 Sept	12	13	10

A total of 19 to 23 experimental manipulations were established per site. Small mussels (25–30 mm) with minimal erosion (<20%) were collected from low-elevation mussel beds (donor beds) near each transplant site (Supplementary Fig. S1). Individual experimental manipulations contained two mussels: one live mussel set in epoxy in an orientation that allowed the shell to continue to open and close, and one mussel that was killed by shucking, which was placed with one half of the empty shell, the exterior of the shell facing outward, set into the epoxy. Experimental manipulations were intentionally spaced over a continuous elevation gradient at each transplant site, spanning the extent of the natural mussel bed, not at fixed intervals (Supplementary Fig. S1). The intent was to capture natural abiotic variation associated with elevation across the extent of each mussel bed. The experimental manipulations were established over a range of dates from April to June 2017 (Table 1) because of the logistical challenges in accessing the intertidal zone at remote sites.

### Mussel and erosion data collection

To test the hypothesis that live mussels are less susceptible to erosion by endolithic cyanobacteria, we quantified shell erosion on every live and empty mussel shell pair at the start and end of the experiment. Pairs of transplanted shells were photographed during the establishment of manipulations (April–June 2017) and at the conclusion of the study (September 2017). The eroded area of the upper mussel shell and the total area of the upper mussel shell were measured from photographs using ImageJ 1.51n (Schneider et al. 2012). We used this datum to calculate the total percentage of shell area eroded during the experiment (see Supplementary Fig. S2). The erosion rate was calculated by measuring the total change in eroded shell area from the start to the end of the experiment and then averaged to a weekly rate (change in proportion eroded*week^−1^). Mussel shell length was measured in the field and used to calculate the growth rate (mm*week^−1^). The growth rates demonstrated that live transplanted mussels maintained normal growth during the experiment (Gehman and Harley 2019).

### Bacterial community sample collection

Samples for epibacterial community analysis were taken in September 2017 (Table 1) at the conclusion of the study, after time for shell erosion by endoliths. We sampled shells *in situ* for 16S rRNA gene amplicon sequencing from each transplanted mussel pair and from unmanipulated mussels at the lowest extent of the donor bed (live controls) at every site. Sampling involved rinsing each shell with 0.22 µm filtered, sterile seawater for 10 s to remove transient environmental microbes and then rubbing with a Puritan® sterile swab for 10 s. Swabs were deposited in individual 2-ml cryovials (VWR), placed in coolers on ice and, upon return to the laboratory (≤6 h after collection), were stored at –80˚C until DNA extraction.

### Abiotic measurements

Elevation in the intertidal zone was calculated using tide charts, combined with measures of seawater height and the height of each manipulation against the stationary position of a laser level. On Calvert Island, elevation and tide height were measured using a combination of Real Time Kinematic (RTK) positioning survey and drone-based digital surface elevation models. Using a geographic information system, elevation data were extracted for all survey areas and temperature loggers not directly measured with the RTK survey. Elevation was calculated via the Canadian system, which uses the Lowest Normal Tides as chart datum (Forrester 1983).

Roughly one-half of the experimental manipulations at each site were inlaid with iButton temperature loggers that recorded the temperature hourly during the study. Temperature data were extracted from each iButton and separated into measurements of exposed substratum (subaerial) and seawater (immersed) based on whether the manipulation was submerged at the time of that measurement using tide charts and the elevation (tide height) of the manipulation. For each experimental manipulation, the mean and 90th quantile temperature values were calculated for subaerial and immersed time periods (Supplementary Fig. S3).

### 16S rRNA gene amplicon sequence processing

DNA was extracted from swabs using the MoBio PowerSoil®-htp 96-well DNA extraction kit (Carlsbad, CA, USA) following the manufacturer's recommended protocol. Extracted DNA was sent to Integrated Microbiome Resource (IMR), Centre for Comparative Genomics and Evolutionary Bioinformatics (CGEB) at Dalhousie University for PCR amplification and library construction. Primers targeted the V4-V5 region of the 16S rRNA gene for bacteria and archaea, namely, 515f: 5′–GTGYCAGCMGCCGCGGTAA–3′ and 926r: 5′–CCGYCAATTYMTTTRAGTTT –3′ (Comeau et al. 2011, Parada et al. 2016). Amplicon library preparation and sequencing with Illumina MiSeq using paired-end (2 × 300 bp) v3 chemistry was performed at the IMR at Dalhousie University, Halifax, Nova Scotia, Canada, according to published protocols (Comeau et al. 2017). Quality filtering, trimming, dereplication, chimera removal, inference of true amplicon sequence variants (ASVs) and taxonomic assignment against the SILVA database (v. 1.3.2) was carried out with DADA2 (Callahan et al. 2016). For DADA2 processing, the filter and trim step was set to a minimum read length of 150 bp forward and 120 bp reverse. Reads were truncated after a quality score of less than or equal to two. Reads with higher than eight forward and 10 reverse maxEE "expected errors'' were discarded. Chimera detection was performed using the pooled method. Singletons and reads assigned as mitochondria were removed for downstream analyses. Of the experimental manipulations sampled, 10 to 12 transplanted pairs per site had sufficient amplicon sequencing read coverage for paired bacterial community analysis. Data from manipulations where only one of the experimental pair had sufficient read coverage were included in all downstream statistical analyses, except those explicitly comparing differences in the composition and relative abundance of taxa between live and empty shells of each experimental pair.

### Statistical analyses

All statistical tests were conducted in R (R Core Team, 2021; version 4.1.2). 16S amplicon sequencing data were rarefied to 8500 reads per sample (Supplementary Fig. S4) using the Phyloseq package (McMurdie and Holmes 2013). To examine the community structure and relative abundance of putative shell-boring cyanobacterial taxa, we analyzed the relative abundance of reads assigned to the phylum Cyanobacteria in experimental treatments and geographic sites, after excluding chloroplast sequences. 16S rRNA gene amplicon sequences from the chloroplasts of microalgae and macroalgae are assigned to the phylum Cyanobacteria. We analyzed the subset of ASVs assigned as chloroplasts separately in an attempt to characterize eukaryotic algal colonizers on mussel shells, which can also be endolithic (Palinska et al. 2017). The relative abundance of cyanobacteria reads, or chloroplast reads, was calculated as the percentage of total reads for each subset of rarefied data. To test for significant differences in the relative abundance of cyanobacterial or eukaryotic algal sequencing reads on live, empty or live control mussel shells, we used Kruskal–Wallis tests. We subsequently conducted these tests for each geographic location independently to look for site-specific differences. We also analyzed the combined datasets (i.e. all ASVs assigned to the phylum Cyanobacteria), to look for patterns common to all potentially photosynthetic taxa.

We used IndVal analysis from the indicspecies package (Cáceres and Legendre 2009) to identify ASVs that were significantly associated with experimental transplants that differed in shell erosion. The IndVal analysis assesses the relationship between ASV occurrence or abundance values from a set of samples and the membership of those samples to groups, which may represent habitat types, sampling points and experimental treatments, etc. The method calculates an IndVal index value based on specificity, or the proportion of samples in a group to which the ASV belongs, and fidelity, or the proportion of the counts of that ASV (abundance) that is exclusive to the group (Dufrêne and Legendre 1997). An index value is calculated for every ASV, in every group, and the ASVs with the highest association value for a particular group are identified as significant using permutation tests. We also used IndVal analysis to identify taxa significantly associated with transplanted versus live control hosts, to characterize any general effects of the experimental manipulation on the prevalent and abundant epibiotic shell bacteria.

To test the hypothesis that bacterial communities on live mussel shells are unique in taxonomic structure compared with empty mussel shells, we used PERMANOVA on Bray–Curtis dissimilarity with sampling location as strata. All PERMANOVA tests were conducted with 999 permutations using the vegan package (Oksanen et al. 2019). Differences in bacterial alpha diversity between live and empty mussel shells were quantified with paired Wilcoxon tests for each of the four sites.

To understand natural geographic variation in epibacterial communities, we compared the alpha diversity between live controls from each of the sites with Kruskal–Wallis tests for both richness (total observed ASVs) and Shannon–Weaver index (H’). We analyzed the Bray–Curtis dissimilarity of live control mussels using PERMANOVA to determine if epibacterial community structure differed significantly by geographic location. We conducted pairwise comparisons between sites using the pairwise Adonis wrapper function (Martinez Arbizu 2020).

The envfit function in the vegan package was used to assess the relationship between geographic location, shell erosion and measured abiotic variables with the first two ordination axes of the PCoA plot of epibacterial beta diversity (Bray–Curtis). The significance of the relationship, based on multiple regression between variables and ordination axes, was assessed with permutation tests. For this analysis, only the subset of the amplicon sequencing data with corresponding iButton temperature data was included. A Mantel test was used to confirm the statistical significance of correlations between continuous abiotic variables and epibacterial community dissimilarity.

Raw 16S rRNA sequence files are available from the European Nucleotide Archive EMBL-EBI database under project accession PRJEB51984. R code for sequence processing and statistical analyses is available on GitHub: https://github.com/katherine-m-davis/Mussel_shell_microbiome/.

## Results

We sampled the epibiotic bacterial assemblages on 45 live unmanipulated control and 135 experimentally transplanted mussels across four geographic sites in British Columbia, Canada (Table 1). A total of 1 530 000 sequence reads belonging to 5733 unique ASVs were kept after processing via the DADA2 pipeline.

### Live mussels experience less erosion by endolithic cyanobacteria

**Figure 2.**
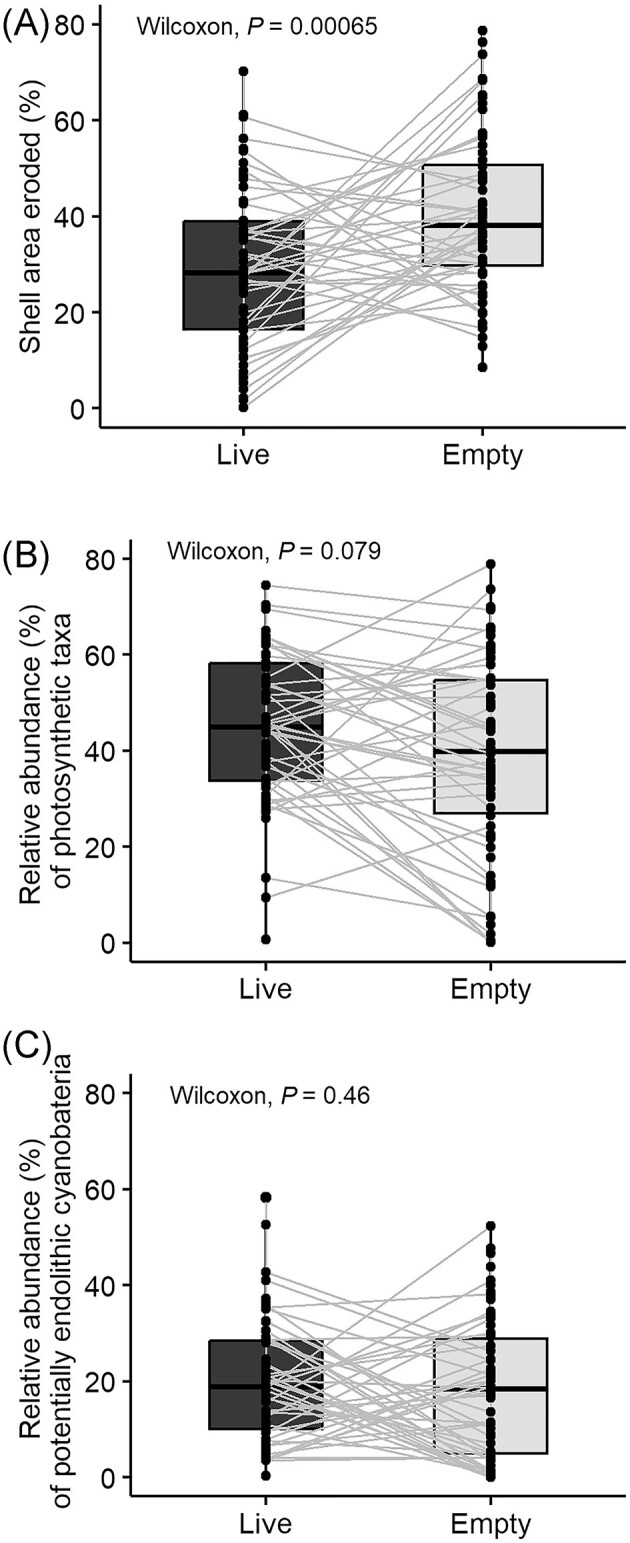
Paired Wilcoxon tests examining differences between experimental pairs of transplanted live mussels and empty mussel shells in (A) percentage of shell area eroded, (B) relative abundance of 16S rRNA reads from all cyanobacteria and eukaryotic algae, and (C) relative abundance of 16S rRNA reads from potentially endolithic taxa.

**Figure 3.**
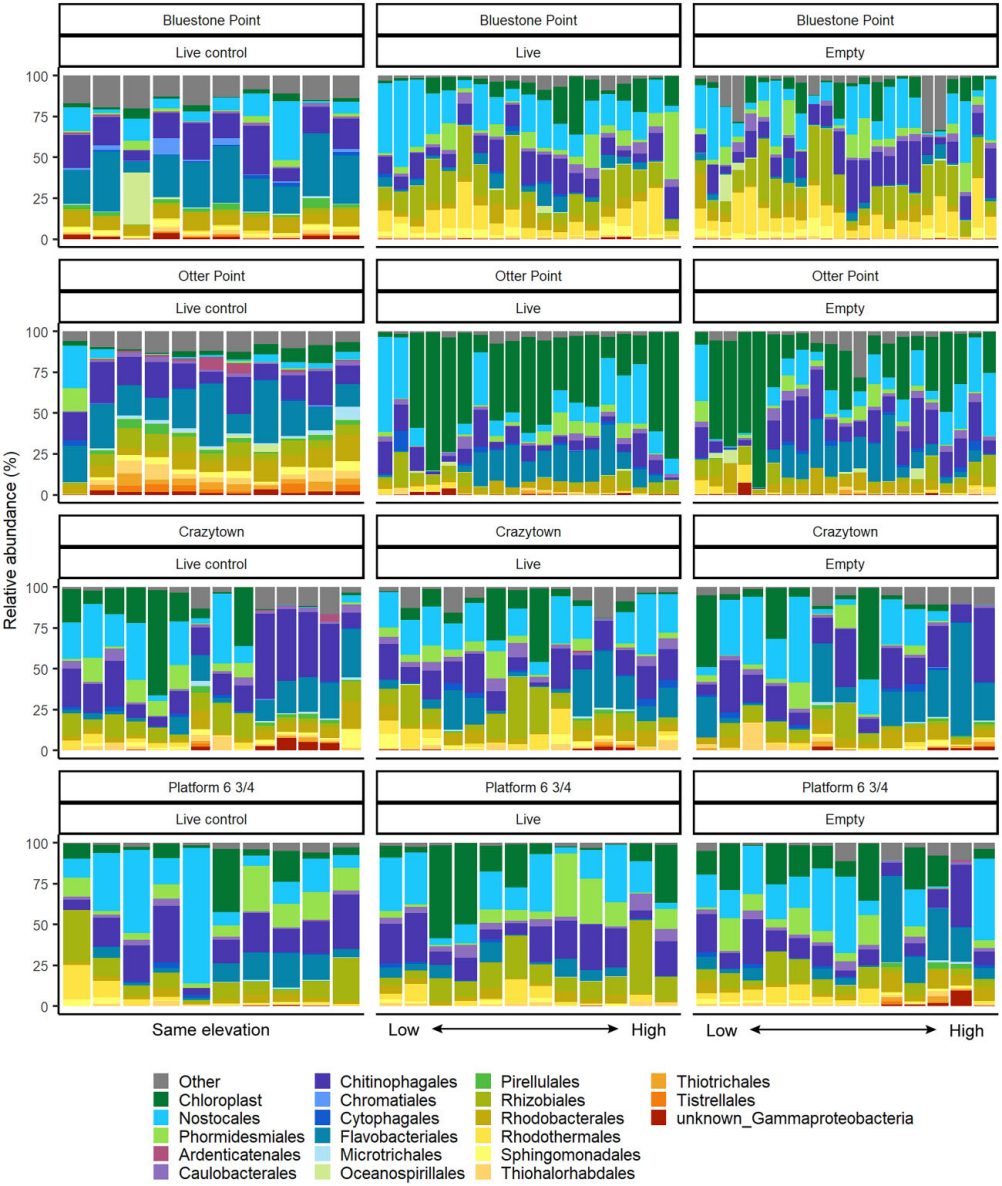
Bar plots of the relative abundance of dominant bacterial families, cyanobacterial families and chloroplast sequences on mussel shells across sites and treatments. Each bar represents an individual mussel shell sample. Samples from live and empty transplants are arranged from left to right by increasing elevation in the intertidal zone. Samples from live control mussels were all taken from the lowest extent of the donor mussel bed at each site.

We tested whether live mussels modulate erosion caused by endolithic cyanobacteria at four rocky intertidal sites. The area of mussel shell eroded over the course of the experimental period was significantly lower for live transplants compared with transplanted empty shells (Wilcoxon *P* = 0.00065; Fig. 2a). Variation in erosion between experimental treatments was driven by significant differences at Otter Point (*P* < 0.001) and Crazytown (*P* = 0.031) (Supplementary Fig. S5). We used IndVal analysis to identify bacterial taxa differentially associated with live and empty mussel transplants that experienced significant differences in erosion, using only samples from Otter Point and Crazytown. Empty mussel shells with increased erosion were more likely to be colonized by uncharacterized Rhodobacteraceae and *Maribacter* spp. (Supplementary Table S1). Live mussel transplants with significantly less erosion were associated with *Maribius* spp. (Supplementary Table S1). The relative abundance of reads from photosynthetic taxa (cyanobacteria and eukaryotic algae) did not differ between live and empty transplants (*P* = 0.079; Fig. 2b and Fig. 3), even among shells that differed in erosion.

### A few taxa dominate cyanobacteria assemblages on mussel shells

We characterized the cyanobacterial assemblages on shells of *Mytilus californianus* to understand the composition and distribution of these organisms in coastal British Columbia (Supplementary Fig. S6). We found the relative abundance of cyanobacteria reads was higher on transplants compared with live controls at the two sites on Vancouver Island (Supplementary Fig. S6), but there was no difference in the relative abundance of cyanobacteria reads between live and empty shells across the dataset (*P* = 0.31). There was no significant association between overall relative abundance of reads assigned to cyanobacteria and mussel shell erosion or elevation in the intertidal zone (*P* = 0.93 and *P* = 0.98, respectively; Supplementary Fig. S7). The cyanobacteria assemblages were dominated by reads from two genera, *Pleurocapsa* and *Phormidesmis*, which consistently co-occurred at roughly the same ratio across the dataset (Supplementary Fig. S6). In a few samples, *Pleurocapsa* was detected but *Phormidesmis* was not. The relative abundance of reads assigned to *Pleurocapsa*, a confirmed endolithic cyanobacterium, did not differ between live and empty mussel transplants (Fig. 2c) and was not significantly correlated with elevation in the intertidal zone (Pearson's R, R^2^ = 0.059, *P* = 0.5). *Phormidesmis* is not endolithic. ASVs assigned to the genera *Leptochromothrix, Pseudophormidium* and *Leptolyngbya* (family Phormidesmiales; also a confirmed endolithic cyanobacteria) occurred at low relative abundance (<3% of the total community), but comprised most of the other cyanobacteria reads detected (Supplementary Fig. S6).

The 16S rRNA gene primers amplify chloroplasts from eukaryotic algae, so we examined these communities on mussel shells because some marine eukaryotic algae are endolithic (Marcelino and Verbruggen 2016). We found chloroplast reads were significantly higher on experimental transplants compared with live controls (Supplementary Fig. S6) and exhibited a significant negative correlation with tidal elevation (Supplementary Fig. S7), but we did not detect any known endolithic taxa.

### Mussel shell bacteria are associated with abiotic factors more than host–parasite interactions

**Figure 4.**
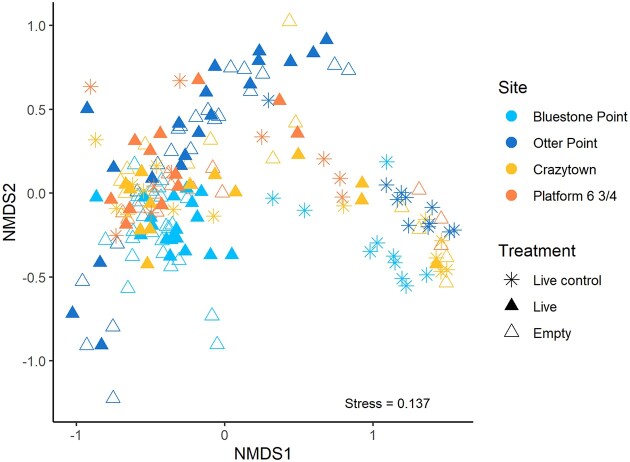
Non-metric multidimensional scaling analysis of Bray–Curtis dissimilarity among epibacterial communities on mussel shells by experimental treatment and site. Corresponding PERMANOVA results comparing treatment groups are presented in Table 2.

We examined patterns of beta diversity across all four sites to understand the contribution of geographic location to variation in the mussel shell bacterial community. Site significantly structured the bacterial communities on shells of unmanipulated live control mussels (PERMANOVA: R^2^ = 0.200, pseudo-F = 3.49, *P* = 0.001; Supplementary Fig. S8) and pairwise comparisons showed significant differences in composition among all sites, except for between Crazytown and Platform 6 & 3/4 (Supplementary Table S2). Alpha diversity, as quantified by ASV richness and Shannon–Weaver index (H’), was significantly higher in mussels from the donor beds on Vancouver Island compared with those from Calvert Island (ASV richness: F = 43.22, *P* < 0.001; H’: F = 27.19, *P* < 0.001). Site also explained significant variation in bacterial beta diversity of experimentally transplanted mussels (PERMANOVA: R^2^ = 0.079, pseudo-F = 5.059, *P* = 0.005). Experimentally transplanted live and empty mussel shells did not have significantly different bacterial communities in terms of alpha diversity (Shannon–Weaver index (H’): *P* = 0.23; ASV richness: *P* = 0.81) (Fig. 3). Beta diversity based on Bray–Curtis dissimilarity was also not significantly different between transplanted live mussels and empty mussel shells (Fig. 4; Table 2). The bacterial community structure on transplanted shells, regardless of host viability, was significantly different from shells of live control mussels (PERMANOVA: R^2^ = 0.040, pseudo-F = 7.506, *P* = 0.001; Table 2; Fig. 3 and Fig. 4). These patterns of beta diversity were consistent with and without the inclusion of chloroplast sequences. The same patterns were also present when considering only manipulations that were transplanted to low elevations, closer to the unmanipulated live controls (Supplementary Fig. S9; Supplementary Table S3). IndVal analysis identified many ASVs, including those assigned to *Litorimonas, Lewinella*, undescribed Rhizobiaceae, *Pleurocapsa* and some chloroplast ASVs, as significantly enriched on the shells of transplanted mussels compared with live controls (Supplementary Table S4).

**Table 2.** Pairwise PERMANOVA results for Bray-Curtis dissimilary between epibacterial communities on mussel shells by experimental treatment. *P*-adjusted values in bold, corrected by the Bonferroni procedure, indicate statistical significance (*P*-adjusted < 0.05).

Treatment comparison	Df	SumsOfSqs	F Model	R^2^	*P*	*P*-adjusted
Empty vs. Live	1	0.35	1.01	0.008	0.4	1
Empty vs. Live control	1	2.22	6.01	0.05	0.001	**0.003**
Live vs. Live control	1	2.58	7.26	0.064	0.001	**0.003**

To understand the effect of abiotic conditions that vary within the intertidal zone on the mussel shell bacterial community, we tested for differences in diversity metrics across tidal elevations of transplanted mussels. There was a significant negative relationship between alpha diversity and tidal elevation at Otter Point for both live and empty transplants (Supplementary Fig. S10), where the tidal elevation range of transplants was greatest. There was no significant relationship between alpha diversity and elevation at any other sites. The relationship between bacterial beta diversity and tidal elevation of transplants was significant across the dataset (PERMANOVA: R^2^ = 0.047, pseudo-F = 6.64, *P* = 0.005), indicating that epibacterial beta diversity on mussels at the same tidal elevation was similar both within and among sites. Because of unequal replication and range of tidal elevations at each site, we also analyzed the relationship between bacterial beta diversity and tidal elevation binned into three categories: low (<2 m), medium (2–3 m) and high (>3 m). After accounting for differences among sites, elevation category was also significantly associated with the bacterial beta diversity of transplants (Supplementary Fig. S9; Supplementary Table S3).

**Figure 5.**
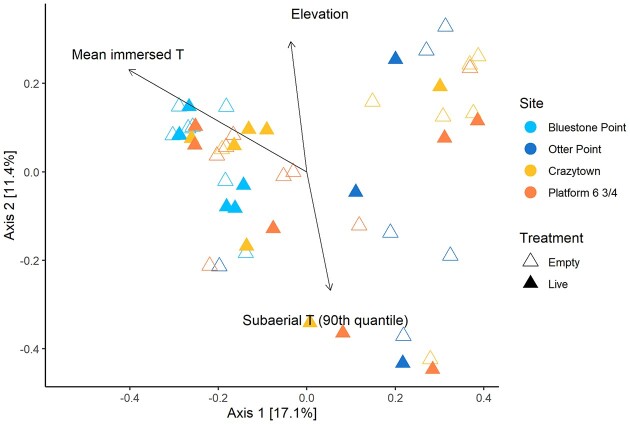
Variables correlated with epibacterial diversity among transplanted mussels. Significant continuous explanatory variables (arrows) from Table 3 are displayed in the PCoA plot based on the Bray–Curtis metric. Plotted points represent dissimilarity values for bacterial communities on transplanted mussels with available iButton temperature **(T)** data.

To further explore the effects of abiotic variation on the mussel shell bacterial community, we examined the relationship between subaerial (substratum) and immersed (seawater) temperatures and epibacterial community composition. Temperature data were collected for about one-half of the transplanted mussel pairs at each site, but there was a significant loss of temperature loggers during the study. Consequently, we examined the subset of transplant pairs with available iButton temperature data (n = 24 pairs; Bluestone Point, n = 6; Otter Point, n = 4; Crazytown, n = 7; Platform 6 & 3/4, n = 7; Supplementary Fig. S3). The variables site, intertidal elevation, mean immersed temperature and 90th quantile subaerial temperature explained significant variation in the first two axes of the multidimensional ordination space for Bray–Curtis dissimilarity of bacterial communities on transplanted mussel shells using envfit analysis (Fig. 5; Table 3). A Mantel test comparing Bray–Curtis dissimilarities and a dissimilarity matrix of elevation confirmed significant dissimilarity of shell bacterial communities between mussel transplants of increasing distance apart along vertical elevation gradients in the intertidal zone (*P* < 0.001). Measured shell erosion was not significantly correlated to elevation of transplants in the intertidal zone (Pearson's R, R^2^ = 0.028, *P* = 0.75), nor was it a significant predictor of epibacterial community composition in the envfit analysis (Table 3).

**Table 3.** Envfit results of the explanatory variables correlated with epibacterial diversity among transplanted mussels. Statistically significant values (*P* < 0.05) are in bold.

	R^2^	*P*
Site	0.243	**0.002**
Treatment	0.018	0.434
Elevation	0.161	**0.015**
Mean immersed temperature	0.393	**0.001**
Subaerial temperature (90th quantile)	0.136	**0.025**
Shell area eroded (%)	0.003	0.945

## Discussion

### Erosion activity by endolithic cyanobacteria depends on host condition

We hypothesized that live mussels would be less susceptible to erosion by endolithic cyanobacteria because live hosts have the potential to influence cyanobacteria colonization and erosion activity by modulating the shell surface. Such modulation could come from active shell surface maintenance, alteration of the shell thermal environment via evaporative cooling, or other biochemical mechanisms. In agreement with our hypothesis, we found significantly less erosion on live mussel shells compared with empty mussel shells from the same transplant pairs. This pattern was driven by significant differences in the percentage of shell eroded between live and empty mussel shell transplants at two out of the four sites. The site-specificity of these results is consistent with findings that endolithic erosion rates can depend on abiotic conditions (Kaehler 1999, Zardi et al. 2009). Alternatively, abiotic factors that varied among sites could have weakened the periostracum and facilitated erosion on empty mussel shells in a site-specific manner (Kaehler 1999). Still, differences in erosion between live and empty mussel shells in some abiotic contexts suggest that live mussels may have the capacity to influence the activity of shell-boring parasites.

Interestingly, we did not find a significant difference in the relative abundance of cyanobacterial reads between live and empty mussel shells at any site. We expected that increased shell erosion would be correlated with a more abundant cyanobacteria community. It is possible that our sampling method did not capture endolithic cyanobacteria cells that were localized inside small bore holes on highly eroded shells, causing us to miss a portion of the cyanobacteria community in the amplicon sequencing data. It is also possible endolithic cyanobacteria colonization and abundance is consistent, but endolithic metabolism differs depending on host condition or site. In such a case, the area of shell eroded would be higher on shells and at sites where cyanobacteria were more active, while the relative abundance of cyanobacteria detected would not differ.

### Known endolithic taxa dominate cyanobacteria assemblages on mussel shells in the northeastern Pacific

The cyanobacteria assemblages on mussel shells were dominated by *Pleurocapsa* spp. and *Phormidesmis* spp. *Pleurocapsa* have previously been described as members of endolithic assemblages and are closely related to endolithic genera, such as *Hyella*, that are responsible for significant shell erosion in other mussel species (Kaehler 1999; Brito et al. 2017; Ndhlovu et al. 2021). *Phormidesmis* are filamentous cyanobacteria with no record of endolithic activity in marine hosts. Co-occurrence of these two taxa at a relatively consistent ratio on mussel shells, and the infrequent observation of *Pleurocapsa* in the absence of *Phormidesmis*, may indicate that *Pleurocapsa* facilitates shell colonization by *Phormidesmis*. We could not explicitly test for such successional dynamics in our study design. It would be an interesting avenue of future research to test this hypothesis using more intensive temporal sampling.

It is not clear why the relative abundance of the putative shell-boring taxa was not positively correlated with measured shell erosion or intertidal elevation as predicted (Kaehler 1999). It is possible that recruitment dynamics of endolithic cyanobacteria might be confounded with the abiotic drivers of erosion activity. Because we selected low elevation mussels with little to no erosion for transplantation, experimental mussels could have had a very reduced endolithic community at the start of the experiment. Cyanobacteria detected on transplanted mussels could have recruited to mussel shells over the course of the experiment. In this case, the microbial samples may have been taken before significant erosion took place under site-specific abiotic conditions. Abiotic factors including water movement and air temperature have previously been shown to affect the distribution and activity of endolithic cyanobacteria (Kaehler 1999). Nonetheless, this study makes an important contribution showing that *Pleurocapsa*, a known shell-boring cyanobacterium, dominates the 16S rRNA sequence reads of cyanobacterial assemblages on California mussels in British Columbia, Canada, which had not been previously characterized using molecular techniques (Bower et al. 2002).

Interestingly, neither of the dominant cyanobacteria we detected on *Mytilus californianus* are common to epibiotic and endolithic assemblages described from other mussel species. The endolithic communities on *M. galloprovincialis* and *Perna perna* are frequently comprised of three to seven species of cyanobacteria from the genera *Hormathonema, Hyella, Kyrthutrix, Plectonema* (*Leptolyngbya*), *Mastigocoleus* and *Solentia* (Ndhlovu et al. 2021, Kaehler 1999, Marquet et al. 2013). We found that sequences assigned to *Leptolyngbya* spp. and *Pseudophormidium* spp. were prevalent, but low-abundance members of the cyanobacterial community on *M. californianus* shells in BC. *Leptolyngbya* spp. are observed as early successional members of endolithic communities on mussels in South Africa (Kaehler 1999, Ndhlovu et al. 2021). They are also recognized for producing diverse secondary metabolites that could potentially influence microbial community assembly on mussel shells (Brito et al. 2015).

It is noteworthy that we detected a high relative abundance of chloroplast reads on mussel shells across the dataset. These include diatoms and macroalgae common at these sites, such as *Ectocarpus* and *Pyropia* spp., and many ASVs with poor taxonomic resolution. Given that marine eukaryotic algae can also be endolithic (Marcelino and Verbruggen 2016, Pernice et al. 2019), it would be interesting to further characterize phototrophic eukaryotic taxa on mussels using more specific molecular markers (18S, ITS, tufA, rbcL) to determine if these organisms are euendoliths, contributing to shell erosion in the northeastern Pacific. It would also be interesting to determine if phototrophic eukaryotic communities on mussel shells are facilitated by endolithic cyanobacteria or other factors (Zuykov et al. 2021).

### Shell erosion and host condition do not structure epibiotic bacterial communities on mussel shells

We hypothesized that increased shell erosion, which can modify host physiology, abiotic conditions experienced by the host (Zardi et al. 2016) and shell microtopographies (Zardi et al. 2009), would influence which bacteria colonize a mussel shell. Our data reject this hypothesis as we did not find a significant impact of increased shell erosion on the composition or structure of mussel shell bacterial communities. It is possible that bacterial community functions, but not the taxa present, may be impacted by the physical habitat modification caused by shell erosion or by the presence of endolithic cyanobacteria (e.g. via photosynthetic exudates). Change in bacterial functions but not taxa has been shown for the *Mytilus edulis* gut microbiome, where carbon metabolism profiles change seasonally, despite consistency in the taxonomic composition (Pierce and Ward 2019). Additional research is needed to understand how bacterial functions are impacted by changes in shell erosion and varying abiotic conditions throughout the intertidal zone.

We tested whether biological filters exerted by live mussels select for different epibiotic bacteria compared with on empty mussel shells. We found no significant difference between the shell bacteria on transplanted live mussels and empty shells. Instead, we found significant differences in the epibiotic bacteria on transplants (live mussels and empty shells) compared with live controls from the donor beds. We also found increased chloroplast reads from microalgae and macroalgae on transplanted mussels compared with live controls. This suggests that transplanted mussels may create new available habitat for macroalgal spores and early bacterial colonizers, and may have experienced reduced competition or grazing compared with on undisturbed, live control mussels. We confirmed that live transplants experienced normal growth (unpublished data) and thus active host biological filters were present in the experimental set-up, suggesting host filtering was not the primary factor shaping epibiotic bacterial assemblages on mussel shells. We note that live control mussels were always from the lowest extent of each mussel bed, while transplanted mussels varied in elevation. Differences in elevation between treatments likely influenced the composition and structure of the bacterial communities sampled.

In a recent meta-analysis, the external microbiomes of marine organisms were found to be more strongly shaped by abiotic conditions than host factors, consistent with our results (Woodhams et al. 2020). Effects of the experimental transplant, for both live mussels and empty shells, also likely produced unintended changes in the local abiotic conditions of transplanted shells, with resulting impacts on the epibiotic community. Embedding transplanted mussel pairs in marine epoxy removed the experimental organisms from dense aggregates, like those in natural mussel beds. As a result, transplants likely received more direct solar exposure and altered hydrodynamics compared with natural, aggregated mussels. Consequently, the temperature and rate at which eroded areas of shell dried out, as well as the seawater circulation and exposure to bacterial colonizers, likely differed between transplanted and control mussels. Further, the epoxy may have temporarily exposed the epibiotic shell community and potential colonizers to chemical leachates. Therefore, the chemical, physical and thermal microenvironments of mussel shells likely differed between transplants and live controls, supporting our finding that the epibacterial community is closely associated with abiotic conditions.

### Correlations between abiotic factors and the bacterial communities on mussel shells

We found differences among sites to be the most significant correlate of bacterial community composition on the shells of unmanipulated control mussels. These results are aligned with observations of strong differences in the bacterial communities of mussels between marine lakes and open marine waters (Cleary and Polónia 2018) and increasing dissimilarity in the gill and shell microbial commmunities of *Mytilus californianus* across a latitudinal gradient (Neu et al. 2021). Community variation across spatial or geographic locations indicates that prevailing environmental conditions and microbial source pools strongly impact bacterial community assembly on mussel shells.

By transplanting mussels across elevations in the intertidal zone, we provide additional evidence for the role of abiotic filtering in shaping the bacterial communities on mussel shells. In particular, we show that elevation, subaerial substratum temperature and immersed seawater temperature all are associated with the observed differences in the mussel shell bacterial community. Intertidal elevation has been shown to influence the digestive gland bacteria of oysters and clams in a transplant study (Offret et al. 2020) and the microbial communities of sympatric macroalgae (Lemay et al. 2020, Quigley et al. 2020). Benthic microbial communities (Rothrock and Garcia-Pichel 2005) are also shaped by geographic location and position in the intertidal zone. We suspect that thermal or other abiotic factors that vary across intertidal gradients can select for bacterial taxa with distinct abiotic preferences (Yung et al. 2015) and lead to spatial community variation in bacteria throughout the intertidal zone. Mussel and oyster gut bacteria have been shown to exhibit temperature-driven compositional convergence at distinct geographic locations (Pierce and Ward 2019). Likewise, the effect of intertidal abiotic gradients is recognized to influence the distribution of macroorganisms (Connell 1972). Our research provides evidence for similar abiotic structuring of bacterial communities on the external surface of an intertidal foundation species. Additional research with greater replication at specific elevations across multiple sites would bolster this finding. The shifts in transplanted mussel shell bacteria observed at different elevations in the intertidal zone here may indicate the potential for future extreme weather events and predicted ocean warming to dramatically impact the mussel shell community and its ecological functions.

In summary, this study provides experimental evidence of increased erosion by endolithic cyanobacteria on empty mussel shells compared with on live hosts. This suggests that host biology may moderate erosion by shell-boring parasites. We also identify potential endolithic cyanobacteria taxa occurring broadly on mussel shells using 16S rRNA sequencing. In contrast to our hypothesis, we show that variation in epibiotic bacterial communities is not associated with the extent of shell erosion or living hosts compared with empty shell. Instead, transplanting mussels along an elevation gradient in the intertidal zone caused marked shifts in bacterial community composition on both live and empty shells. Our findings demonstrate that spatially variable abiotic factors correlate more strongly with epibacterial community structure on the California mussel than biotic interactions with the host or shell-boring parasites. The results from our experimental transplants further indicate that alterations in abiotic conditions have the potential to significantly impact constituents of epibiotic communities on mussel shells, with unknown consequences for host health and ecosystem functions.

## Supplementary Material

fiae101_Supplemental_Files
