# Supplementary material for: Epibiont communities on mussels in relation to parasitism and location in the rocky intertidal zone
Source: FEMS Microbiol Ecol. 2024 Aug 13;100(9):fiae101. doi: 10.1093/femsec/fiae101 (PMC11385189; doi:10.1093/femsec/fiae101)

Supplementary Figure 1. Representative image that shows the natural extent of the mussel bed, the location of the donor/live control samples (black oval), and the location of transplanted experimental manipulations for one site (pink points). A full site overview is represented in the upper right, with closer images indicated by the yellow and green bounding boxes. Elevation of each experimental manipulation is indicated.


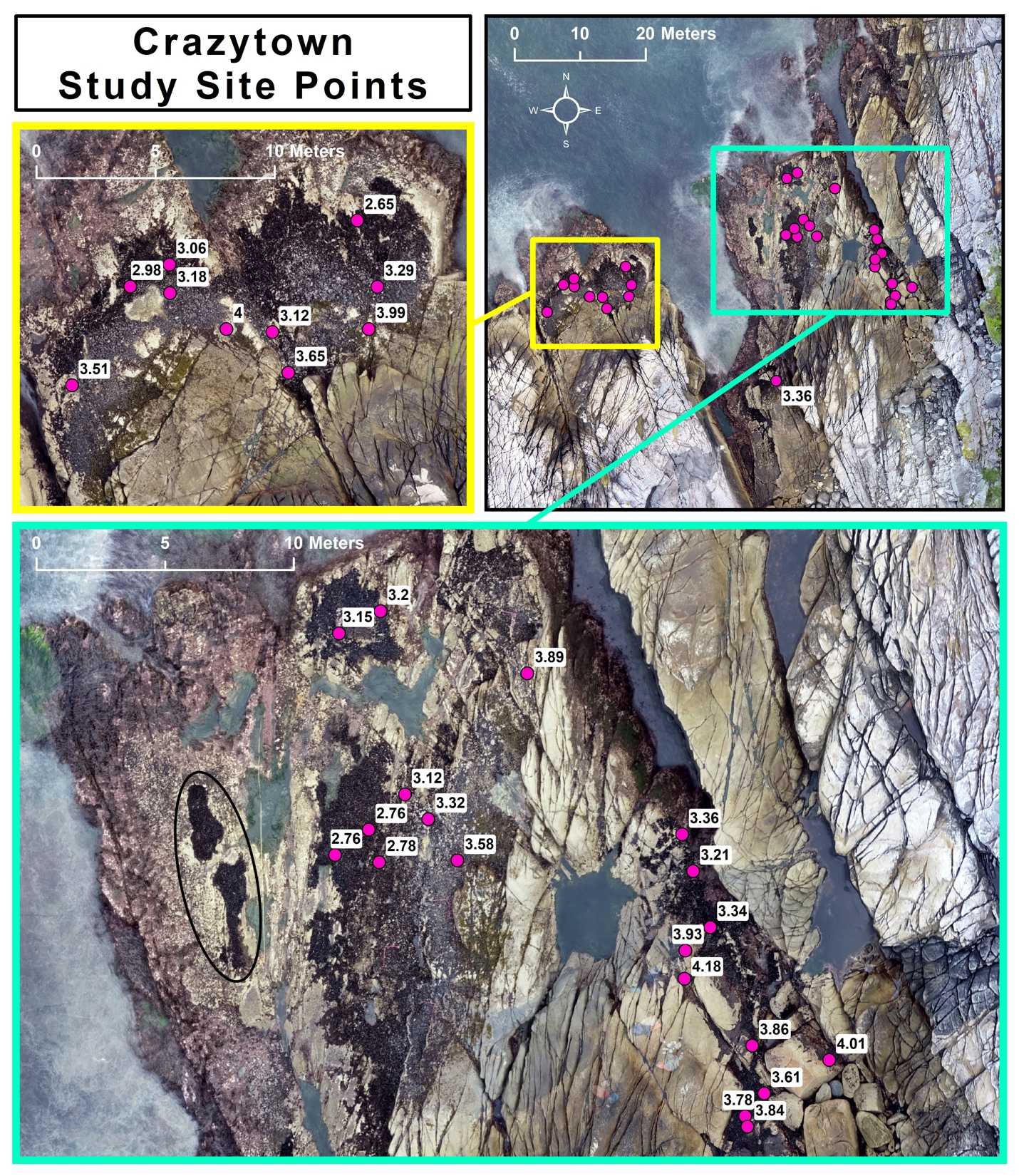


Supplementary Figure 2. Two example images showing how the eroded area of the upper mussel shell and the total area of the upper mussel shell were quantified from photographs and used to calculate the total percentage of shell area eroded during the experiment.


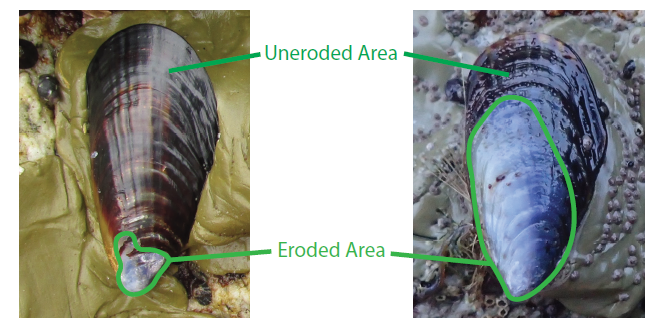


Supplementary Figure 3. Summary temperature data for manipulations with iButton loggers at each site for time periods when loggers were in seawater (immersed) and exposed (subaerial).


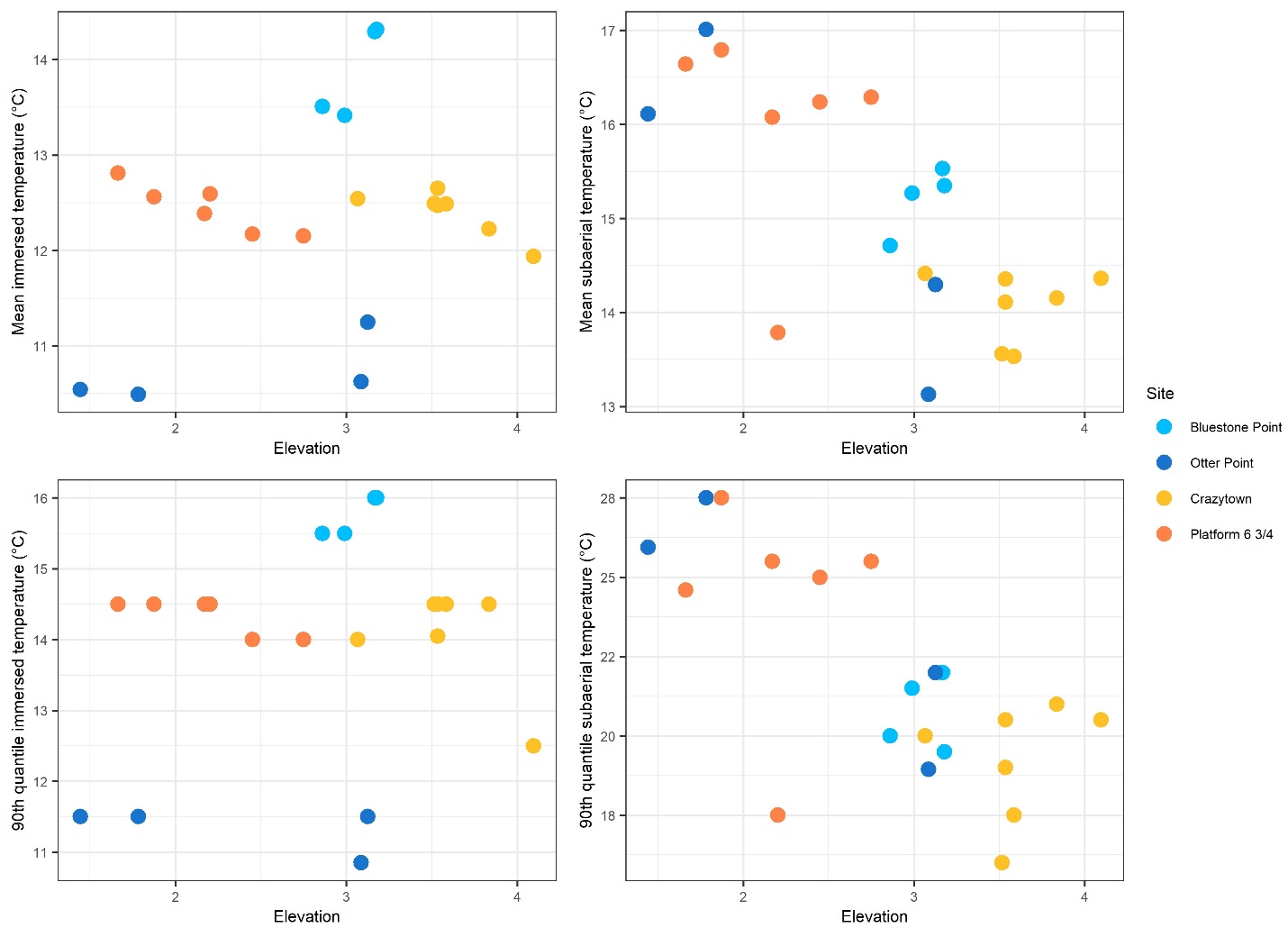


Supplementary Figure 4. Rarefaction curve.


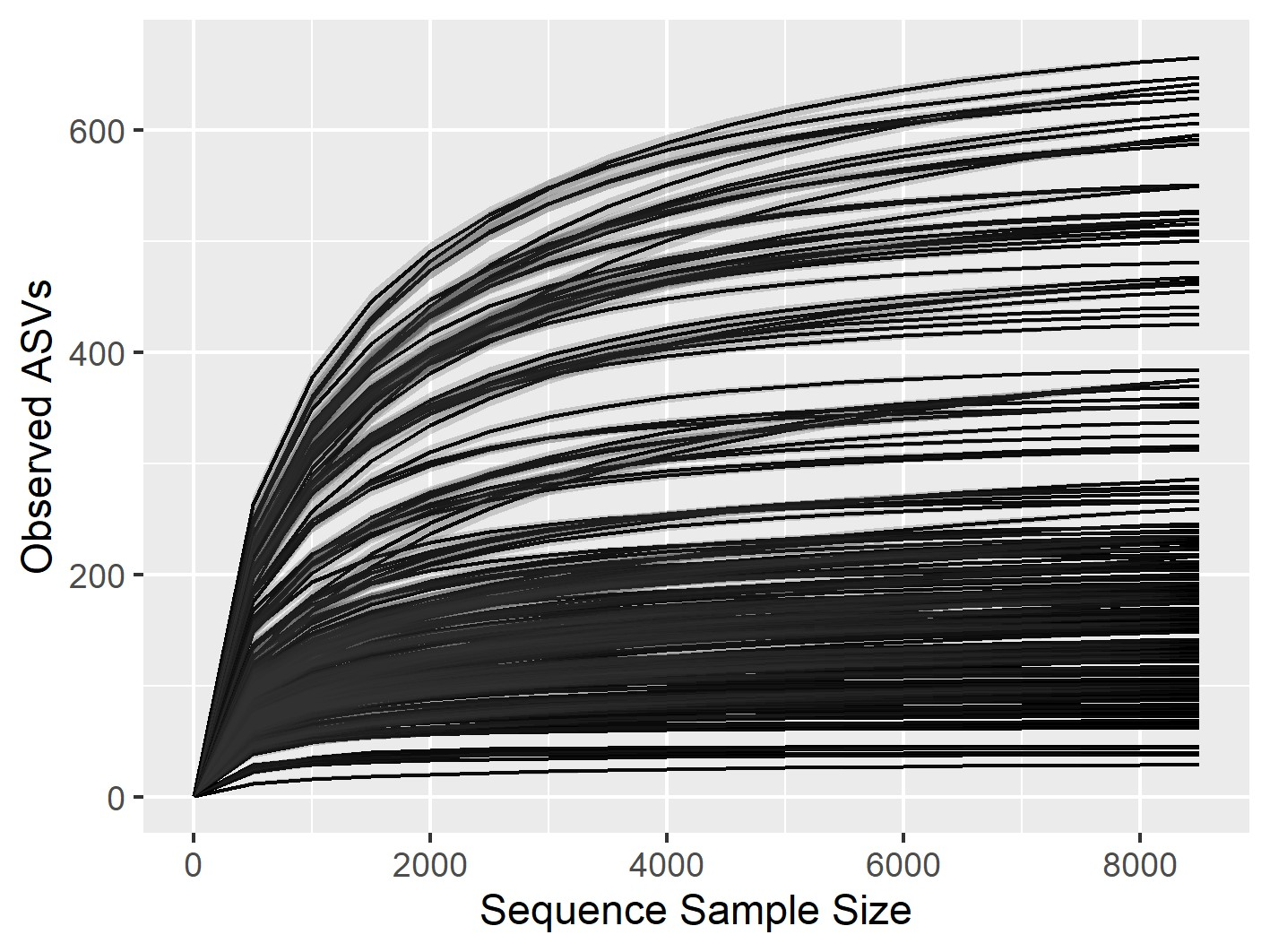


Supplementary Figure 5. Differences in area of shell eroded between transplanted experimental pairs of live mussels and empty shells at each site.


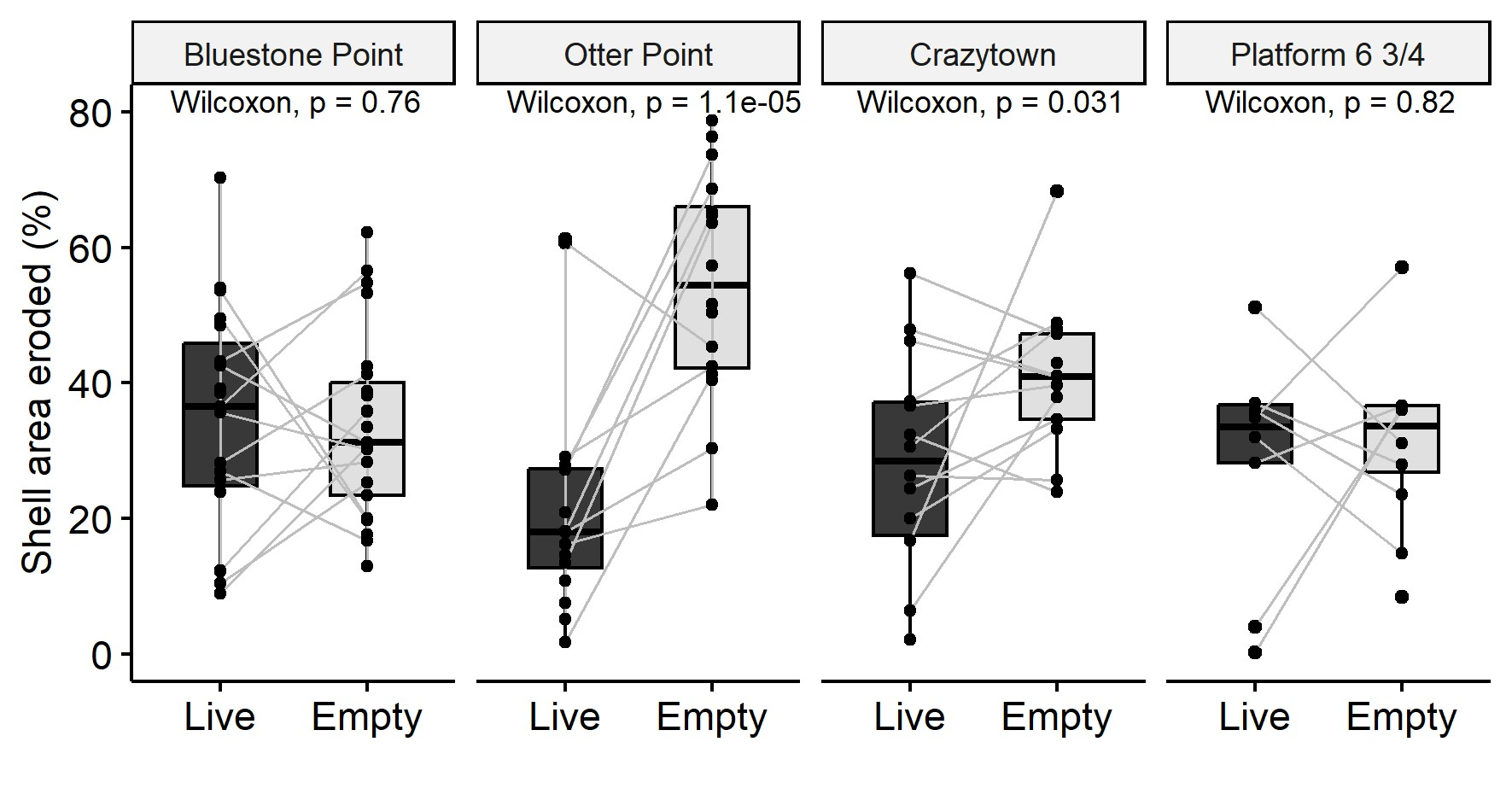


Supplementary Figure 6. Bar plots of the relative abundance of cyanobacteria and algal taxa sequences on mussel shells across sites and treatments. Each bar represents an individual mussel shell sample. Samples from live and empty transplants are arranged from left to right by increasing elevation in the intertidal zone. Samples from live control mussels were all taken from the lowest extent of the donor mussel bed at each site.


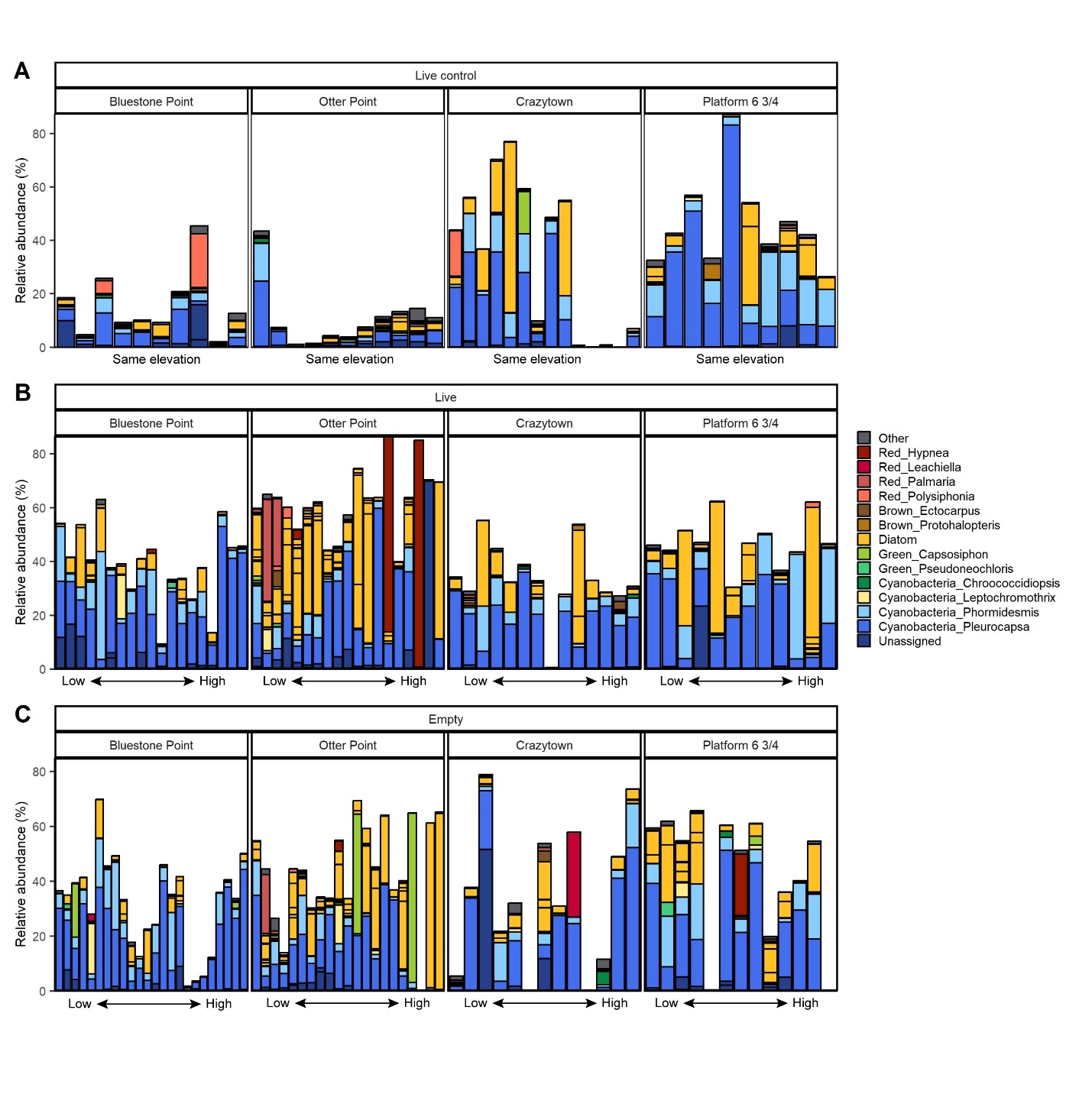


Supplementary Figure 7. Correlations between the relative abundance of sequencing reads assigned to photosynthetic taxa and shell erosion (A,C) or elevation in the intertidal zone from low to high (left to right) (B,D) for cyanobacteria and eukaryotic algae respectively.


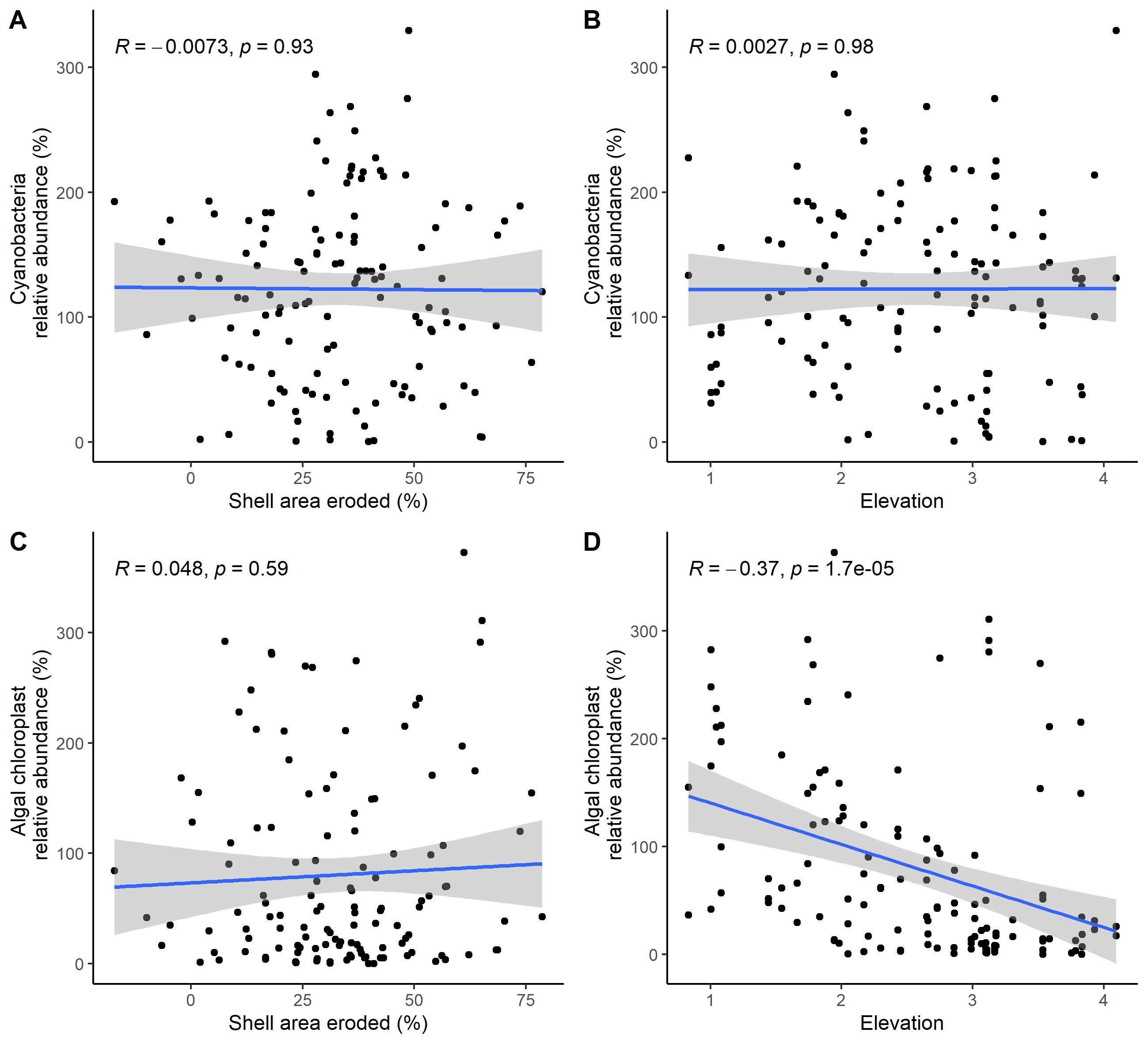


Supplementary Figure 8. PCoA of Bray-Curtis dissimilarity values for epibacterial communities on live control mussels at each site.


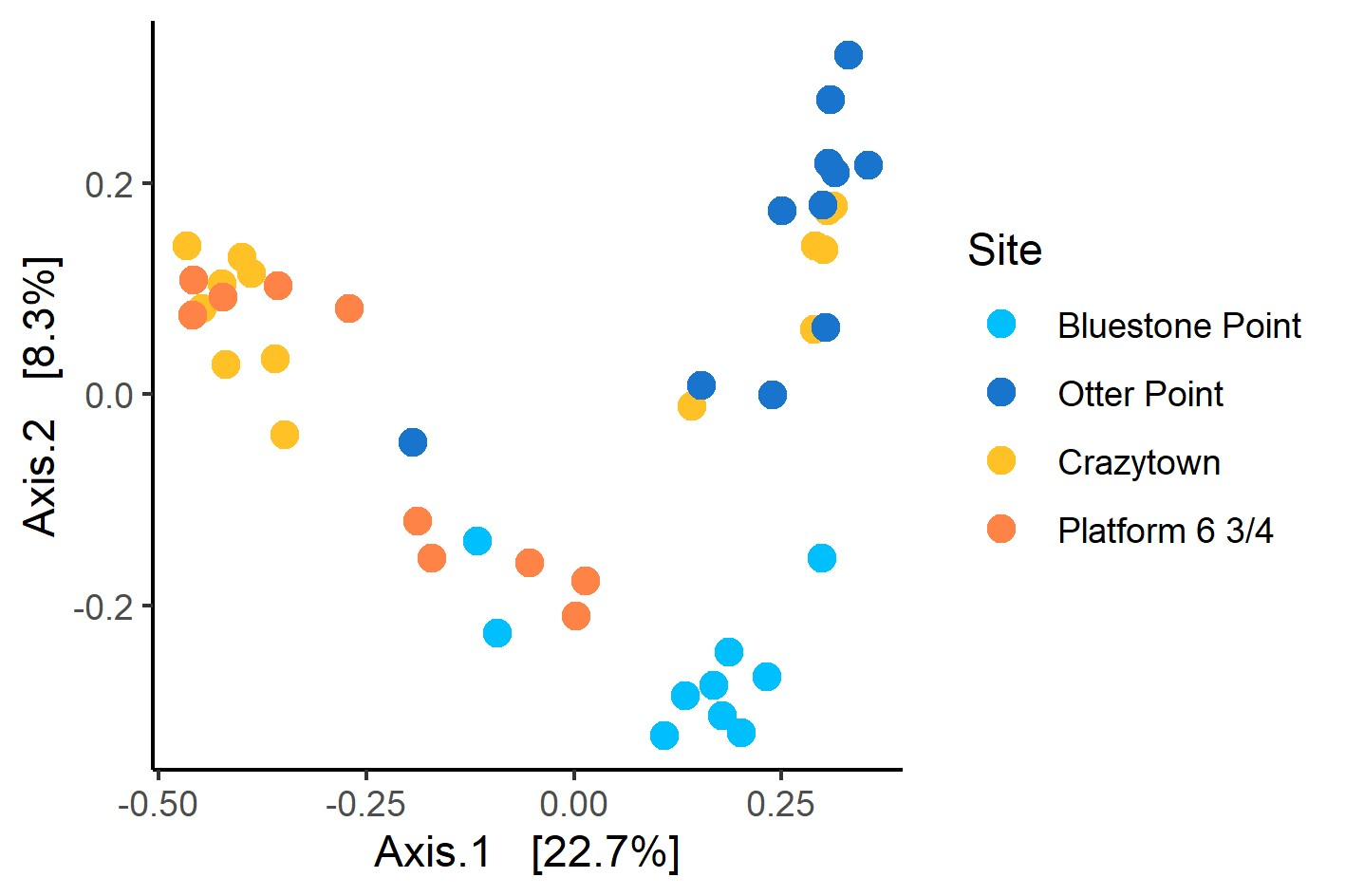


Supplementary Figure 9. A) PCoA of Bray-Curtis dissimilarity values for epibacterial communities on the three lowest elevation transplant pairs compared to the unmanipulated live controls sampled from the lowest extent of the donor mussel bed. B) PCoA of Bray-Curtis dissimilarity values for epibacterial communities on transplant pairs based on the elevation category, low (< 2m), medium (2-3m), high (> 3m), of the experimental manipulations at each site. Corresponding PERMANOVA results for each plot are presented in SI Table 3.


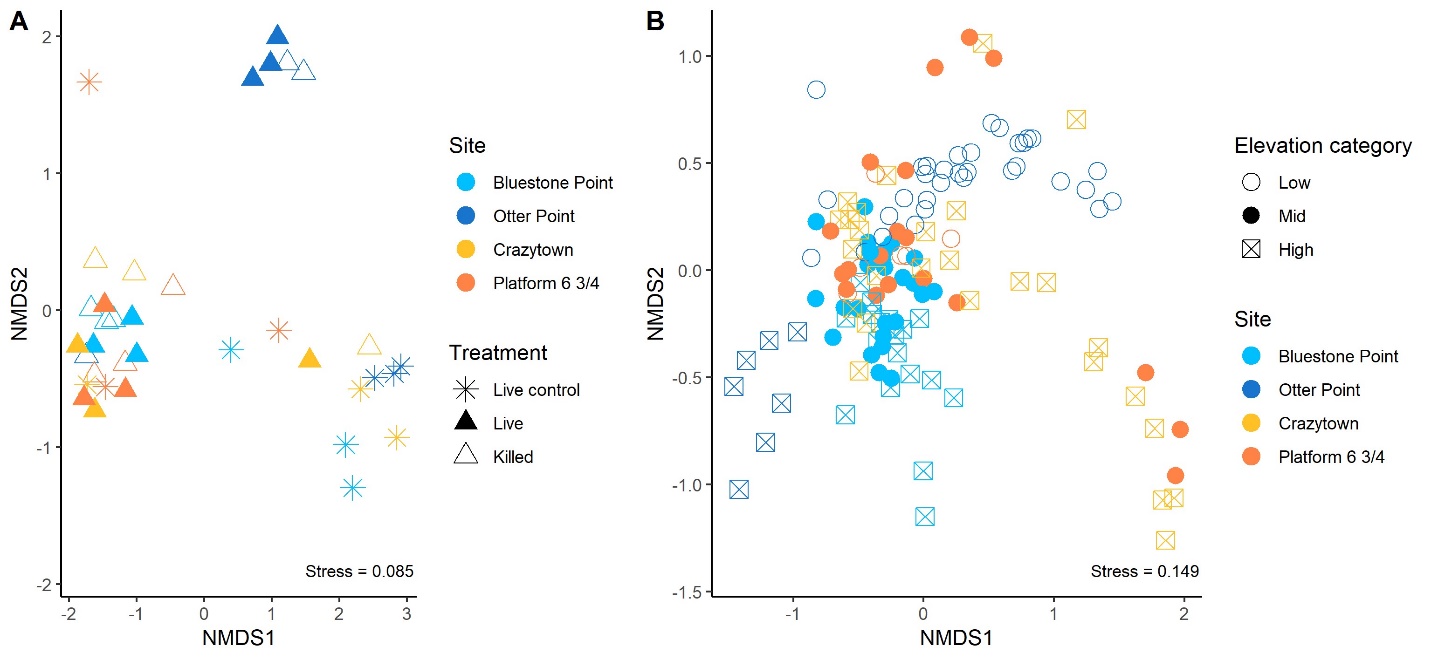


Supplementary Figure 10. Alpha-diversity values for experimental treatments plotted by elevation in the intertidal zone and grouped by site.


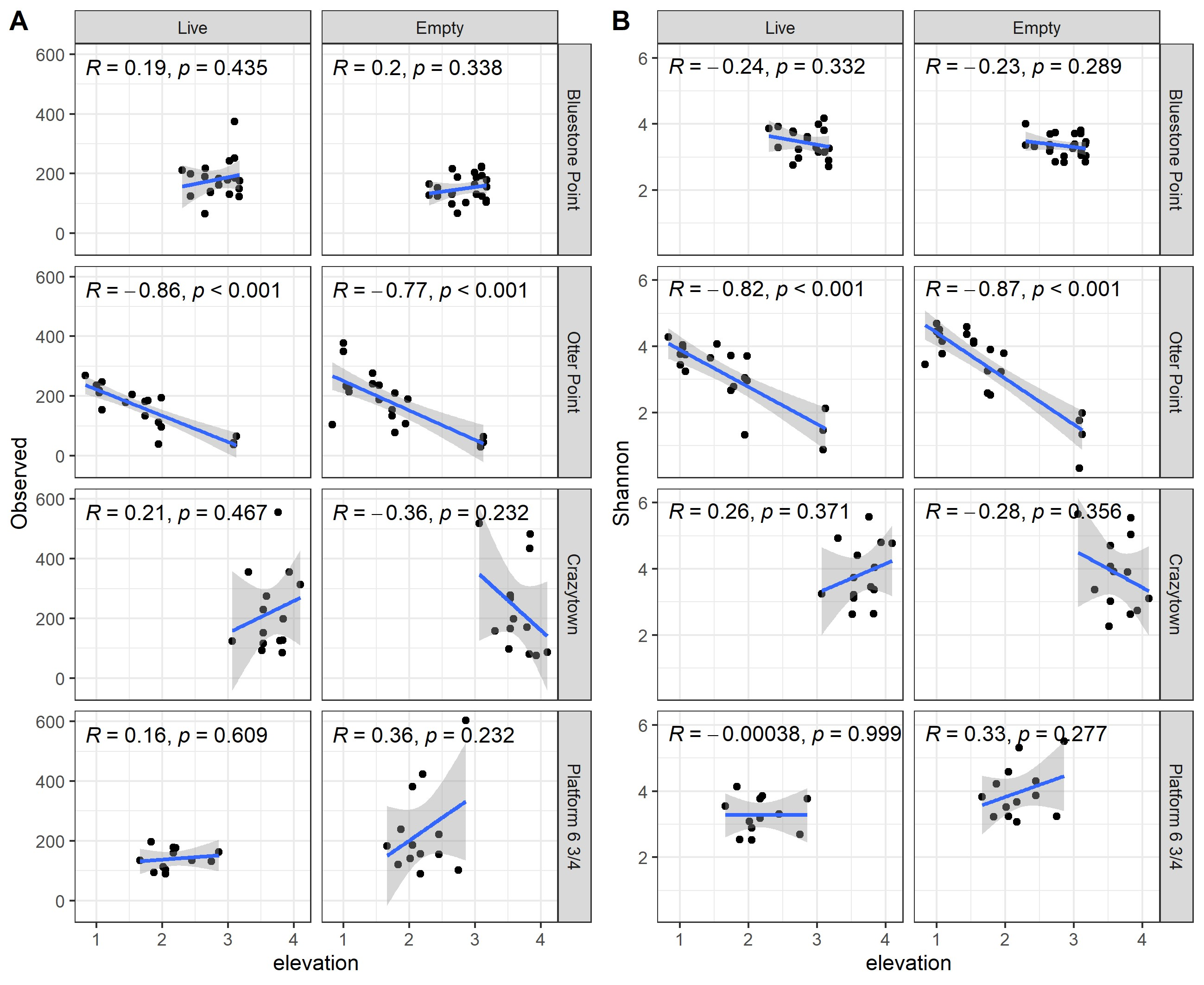

Supplement: fiae101_Supplemental_Files [file fiae101_supplemental_files.zip › Mussel_microbiome_REVISED_Supplementary data_Figures_16-June-2024.docx]
